# Response to intranasal *Lactococcus lactis W136* probiotic supplementation in refractory CRS is associated with modulation of non-type 2 inflammation and epithelial regeneration

**DOI:** 10.3389/falgy.2023.1046684

**Published:** 2023-03-15

**Authors:** Saud Al-Romaih, Oumkaltoum Harati, Leandra Endam Mfuna, Ali Filali-Mouhim, Audrey Pelletier, Axel Renteria Flores, Martin Desrosiers

**Affiliations:** ^1^ Department of Otolaryngology, Head and Neck Surgery, Faculty of Medicine, King Saud University, Riyadh, Saudi Arabia; ^2^ Centre de Recherche du Centre Hospitalier de l’Université de Montréal (CRCHUM), Montreal, QC, Canada; ^3^ Division of Otolaryngology-Head & Neck Surgery, Centre Hospitalier de l'Université de Montréal, Montréal, QC, Canada

**Keywords:** chronic rhinosinusitis (CRS), nasal polyposis, probiotics, microbiome, transriptomic, inflammation, nasal polyps, epithelium, type 1 inflammation

## Abstract

**Justification:**

We have previously documented that in individuals with chronic rhinosinusitis (CRS) refractory to surgery, intranasal application of live *Lactococcus lactis W136*, a probiotic bacterium, improves sinus-specific symptoms, SNOT-22, and mucosal aspect on endoscopy, accompanied by a reduction in sinus pathogens and an increase in protective bacteria. The present work explores the molecular mechanisms underpinning these observations using transcriptomics of the sinus mucosa.

**Method:**

Epithelial brushings collected prospectively as a sub-study of the *L. lactis W136* clinical trial were used to probe epithelial responses to microbiome supplementation using a hypothesis-free bioinformatic analysis of gene expression analysis. Samples from twenty-four patients with CRS refractory to medical and surgical management were prospectively collected during a clinical trial assessing the effect of 14 days of BID nasal irrigation with 1.2 billion CFU of live *L. lactis W136* probiotic bacteria (CRSwNP = 17, CRSsNP = 7). Endoscopically guided sinus brushings were collected as part of the initial study, with brushings performed immediately before and after treatment. Following RNA extraction, samples were assessed using the Illumina HumanHT-12 V4 BeadChip. Differential gene expression was calculated, and pathway enrichment analysis was performed to identify potentially implicated processes.

**Results:**

Differentially identified transcripts and pathways were assessed for the overall population and the clinical phenotypes of CRSwNP and CRSsNP. Patterns of response to treatment were similar across all groups, implicating pathways for the regulation of immunity and epithelial cell regulation. These resemble the patterns of improvement observed following successful treatment with endoscopic sinus surgery or azithromycin.

**Conclusion:**

Gene expression profiling following the application of live bacteria to the diseased sinus epithelium highlights the implication of multiple components of the inflammation-microbiome-epithelial barrier axis implicated in CRS. These effects appear to involve both epithelial restoration and modulation of innate and adaptive immunity, supporting the potential interest of targeting the sinus epithelium and the microbiome as potential CRS therapies.

## Introduction

Sinus cavity homeostasis is ensured by continuous interaction of the epithelial barrier with resident microbiota, which serves to condition not only immune responses but also epithelial differentiation and regeneration. Chronic rhinosinusitis (CRS) is an inflammatory condition of the upper airways of uncertain origin which is characterised by disruption of normal homeostasis with ensuing tissue inflammation, microbiome dysbiosis, and epithelial dysfunction (1) Characteristic changes include infiltration with a variable mixture of Type 2 and Type 1/Type 17 inflammatory cells, and impaired epithelial function and regeneration, accompanied by concomitant microbiome dysbiosis (2). Multiple pathogens have been implicated, but *Staphylococcus Aureus* represents a frequent and problematic pathogen, particularly in CRSwNP and in the elderly population (3). While initial events leading to the development of disease remain to be identified, the persistence of these tissue changes is believed to reflect dysfunction of the three defensive pillars, behaving as an epithelium-inflammation-microbiome axis which ensures the health of the sinus cavity and its epithelial lining.

The emerging concept that successful disease management will have an impact on all three components of the axis, either directly or indirectly *via* downstream effects, suggests that all three components represent targets for CRS treatment. While immune dysfunction has long been the principal target of therapy (5), it is increasingly obvious that targeting other components of the axis, such as the epithelium, can also successfully modulate disease (6) For instance, successful treatment with Elexacaftor-Tezacaftor-Ivacaftor (Trikafta), a new medication used in cystic fibrosis (CF) which restores deficient CFTR expression, is associated with near-complete resolution of CF-related sinus disease, as observed by the improvement in symptoms and restoration of a near-normal epithelial aspect, even in the absence of concomitant antibiotic therapy (7).

Given the success of modulation of epithelial function, modulation of the microbiome may thus represent another strategy, and nasal microbiome supplementation with intranasally administered probiotic bacteria has previously been suggested as a novel means of treating CRS (8, 9). We have previously demonstrated that intranasal administration of *Lactococcus lactis W136* bacteria is well-tolerated and improves symptoms, endoscopic aspect of the mucosa and quality of life in patients with CRS (9). Microbiome-modulating effects were also observed, with an increased abundance of *Dolosigranulosum Pigrum*, a putative beneficial pathobiont recently identified in both adults and children as a bacterial species associated with health in the nose, sinuses, nasopharynx, and adenoid. Subgroup analysis according to clinical phenotype showed a reduction in the abundance of *Staphylococcus aureus* in patients with CRSwNP and a reduction in multiple strains of *Pseudomonas Aeruginosa* in CRSsNP (9).

While these intriguing observations suggest an effect of microbiome supplementation on CRS, the mechanisms underpinning these changes remain to be described. We thus wished to further characterise the molecular mechanisms of these changes at the tissue level by exploring changes in gene expression induced by the probiotic *L. lactis W136* treatment in the sinus mucosa.

## Methods

### Participants and study design

Epithelial brushings collected prospectively as a sub-study of the *L. lactis W136* clinical trial were used to probe epithelial responses to microbiome using a hypothesis-free bioinformatic analysis of gene expression analysis. The *L. lactis W136* for the CRS trial has been described in detail elsewhere (9). Approval was obtained from Health Canada for intranasal administration of live L. lactis *W136* bacteria (Health Canada registration number: 191920) and the CHUM Institutional Review Board and Ethics committee (Registration No. 12.288) prior to the trial performance (*Clinicaltrials.gov* identifier: NCT04048174). Briefly, twenty-four patients with CRS refractory to previous medical and surgical therapy received a 14-day course of BID sinus irrigations containing 1.2 × 10^9^ CFU of live *L. lactis W136*. No patients had received oral corticosteroids or topical or systemic antibiotic therapy in the preceding 30 days. Only saline irrigation was allowed for symptom relief. Excluded were patients <18 years, CF, with technical reasons for endoscopic sinus surgery (ESS) failure, active sinus infection with purulence, pain and/or hyperthermia, or with immune suppression from disease or medication.

Epithelial brushing was collected under endoscopic control using a gastric cytology brush at the level of the frontal recess prior to the first probiotic application at D0, and the day following the last treatment at D14. Total RNA was extracted using RNeasy Mini Kit (QIAGEN, Toronto, On, Canada). Gene expression was performed at Genome Quebec Innovation Center (Montreal, Qc, Canada) using the Illumina HumanHT-12 V4 BeadChip (Illumina, San Diego, CA, United States).

### Gene expression data analysis

Raw Illumina probe data were exported from BeadStudio and screened for quality. Pre-processing and statistical analysis were conducted using the R statistical language and software packages from Bioconductor as described by Huber et al. (10). Quantile normalization was applied, followed by a log2 transformation.

#### Differential gene expression

For each probe, a paired linear model was fitted using the LIMMA package from Bioconductor with a donor blocking factor. The LIMMA package implements a moderated *t*-test used to compare gene expression before and after intranasal administration of *L. lactis* at D0 and D14 respectively. *P*-values from the resulting comparison were adjusted for multiple testing according to the method of Benjamini and Hochberg (11). This method controlled the false discovery rate (FDR), which was set to 0.05.

#### Pathway enrichment analysis

Gene Set Enrichment Analysis (GSEA) was performed using the Bioconductor's package FGSEA using gene sets from the Molecular Signature Database (MsigDB, http://www.broad.mit.edu/gsea/msigdb): Hallmark (h.all.v5.0.symbols.gmt) (12).

GSEA-associated pathways *P*-values were adjusted for multiple test corrections with FDR cut-offs of 0.05. GSEA was performed to assess whether a known biological pathway or sets of individual genes were significantly enriched among the genes ranked by the moderated *t*-test following the differential gene expression analysis.

## Results

**Figure 1:**
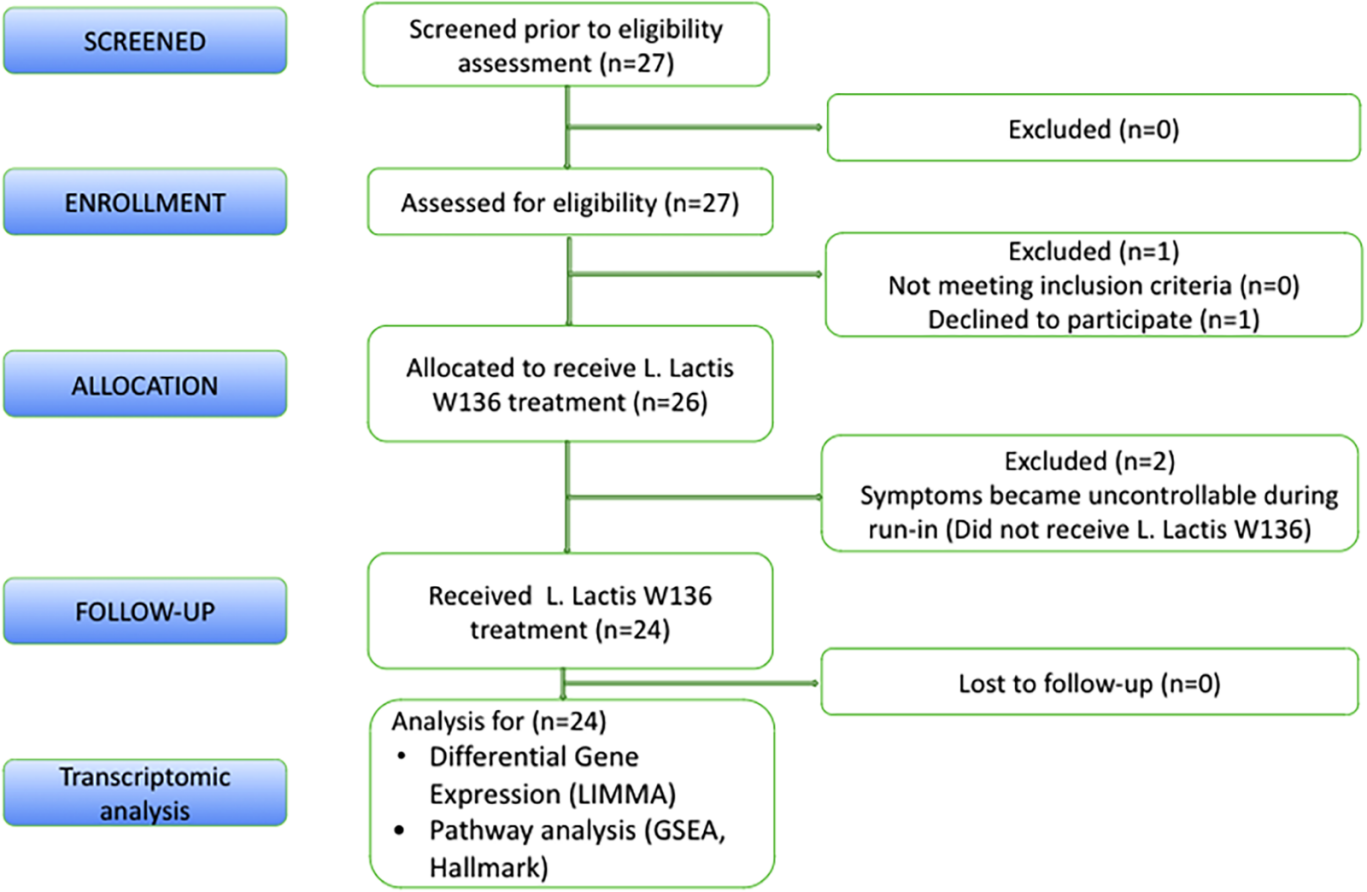
CONSORT statement.

As previously described in the clinical study, all 24 patients receiving *L. lactis W136* completed the study (Figure 1) (9). We have included patients with persistent symptoms and signs of CRSsNP or CRSwNP despite undergoing technically adequate surgery and continued use of maximal medical therapy, including high-volume budesonide irrigations post-operatively (“refractory” CRS). The population baseline characteristics were outlined in Endam et al. (9). Overall, 14 days of treatment with *L. lactis* improved sinus-specific symptomatology, SNOT-22 score, and mucosal aspect on endoscopy, with beneficial microbiome changes. Differential gene expression between pre- and post-treatment conditions was assessed for paired samples only in (i) All CRS (*N* = 20), (ii) CRSwNP subgroup (*N* = 16) (iii) CRSsNP subgroup (*N* = 4) and (iv) Responders (*N* = 10) (as defined by a reduction in SNOT-22 score of 8.9 or greater). The responders included both CRSwNP (*N* = 8) and CRSsNP (*N* = 2) patients. The transcriptomic assessment demonstrated differences associated with treatment. Individual gene transcripts with FDR  ≤ 5% and ≥1.2-fold-change in expression were noted only in the “All CRS” groups and in the CRSwNP subgroup (Table 1 and Supplementary material). Comparatively to CRSwNP, the expression of very few genes were significatively modified after treatment in CRSsNP, presumably as a consequence of the low number of individuals in that subgroup. Gene expression Heatmaps of the top differentially expressed gene (Supplementary Figures S1–S4) reveal similar differential gene expression patterns for all populations, suggesting a common response pattern.

**Table 1:** Differentially expressed genes (DEG) in response to Lactococcus Lactis W136 probiotic treatment.

	DEG Up FDR adjusted *P*-value < 0.05	DEG Down FDR adjusted *P*-value < 0.05	DEG Up non-adjusted *P*-value < 0.01	DEG Down non-adjusted *P*-value < 0.01
**All CRS** Post vs. Pre- treatment	36	19	199	405
**CRSsNP** Post vs. Pre- treatment	0	0	39	16
**CRSwNP** Post vs. Pre- treatment	11	4	180	392
**Responders** Post vs. Pre- treatment	0	0	84	64
Responders vs. Non-responders	0	0	18	68

**Figure 2:**
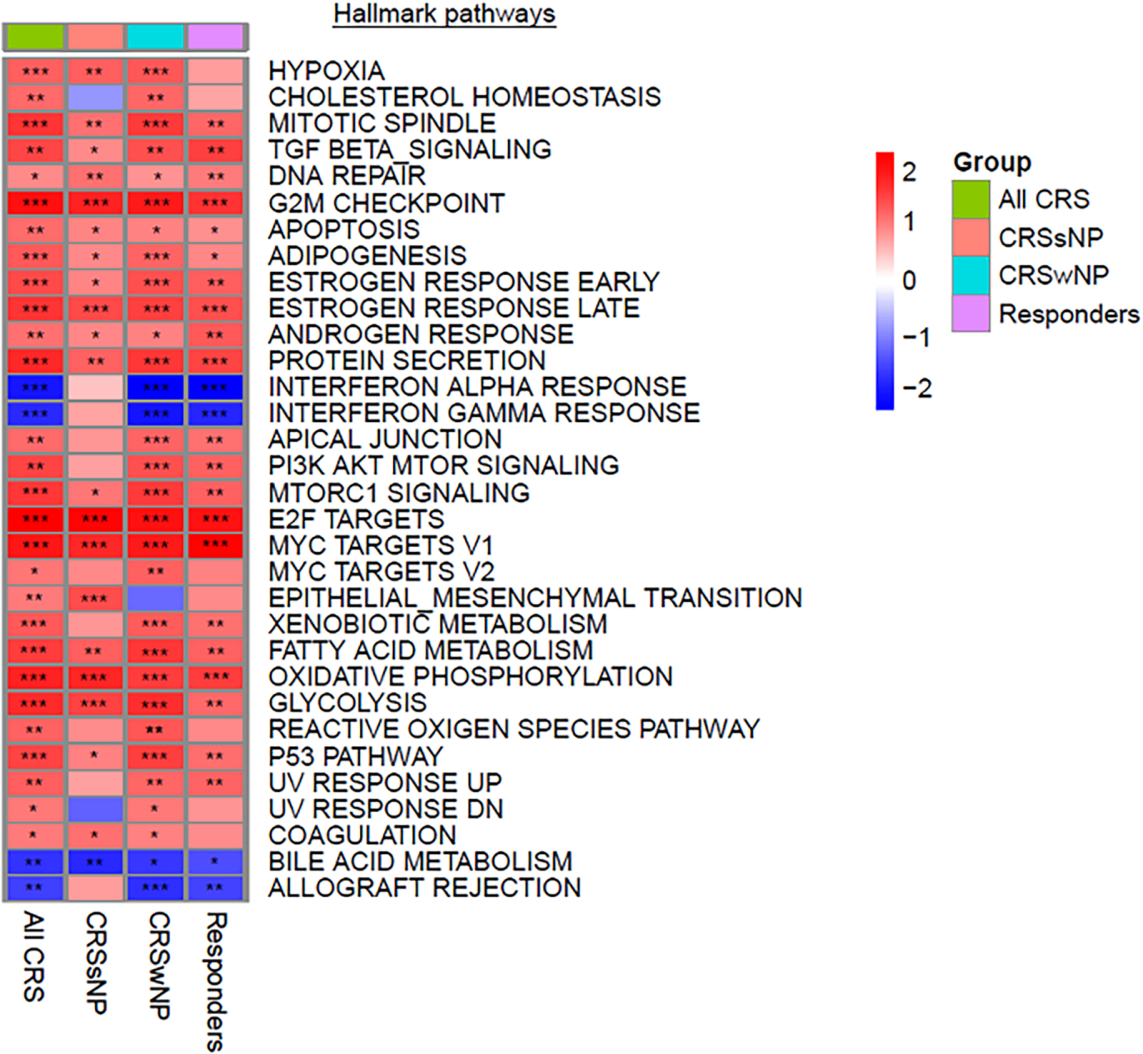
Significantly modulated pathways in both CRSwNP and CRSsNP post vs. pre-treatment with Lactococcus Lactis W136 probiotics. Heatmap of gene set expression analysis (GSEA) hallmark pathways modulated after probiotics treatment. All pathways presented have an FDR < 0.05. Data is presented as normalized enrichments scores (NES) where values >0 represent upregulation and values <0 represent downregulation for each contrast. *P*-values are indicated by stars; *P < 0.1, **P < 0.01, ***P < 0.001.

A pathway enrichment analysis was performed to identify potential underlying mechanisms of response to treatment (Figure 2 and Supplementary Figure S5). Significantly associated pathways were identified for all groups and revealed that treatment with *Lactococcus Lactis W136* induced upregulation of cellular cycle, growth, and proliferation pathways. While enriched hallmark pathways were similar between groups, the differences were more significant in ALL CRS and CRSwNP groups. Notably, a common mechanism appears to involve the upregulation of cell cycle and transcription (E2F targets, epithelial-mesenchymal transition, G2M checkpoint, MYC targets, protein secretion, mitotic spindle), reactive oxygen species (ROS) regulation (hypoxia, ROS pathway, oxidative phosphorylation). The modulation of immunity was also indicated by the upregulation of signalling pathways (PI3K-AKT-MTOR signalling, MTORC1 signalling, TGF beta signalling) accompanied by downregulation of adaptive (allograft rejection) and Type 1-associated immune responses (interferon alpha response, interferon-gamma response). Differential expression in selected significant pathways demonstrates a consistent pattern of enhanced epithelial renewal in response to probiotic treatment. Reinforcement of this transcriptional signature in the population restricted to SNOT-22 responders indicated that these were associated with observed clinical improvements. Pathways were similar between phenotypes of CRSsNP and CRSwNP, suggesting a common mechanism of effect in both populations (Table 2). Identified pathways are loosely grouped into Cell cycle and differentiation, Reactive oxygen species (ROS) regulation, DNA repair, and Inflammation-related pathways. Despite small group sizes hampering analysis, results demonstrate a consistent mechanism of response across groups.

**Table 2:** Significant GSEA pathways in subgroups: (i) All CRS patients, (ii) Chronic sinusitis with nasal polyps (CRSwNP), (iii) Chronic sinusitis without nasal polyps (CRSsNP), (iv) Responders.

	All CRS		Responders		CRSwNP		CRSsNP	
	NES	Adj *P*-value	NES	Adj *P*-value	NES	Adj *P*-value	NES	Adj *P*-value
UPREGULATED HALLMARK PATHWAYS								
APICAL JUNCTION	1.48	0.0073	1.43	0.0183	1.54	0.0024		
APOPTOSIS	1.47	0.0140						
CHOLESTEROL HOMEOSTASIS	1.48	0.0433			1.52	0.0202		
E2F TARGETS			2.38	0.0017	2.39	0.0008	2.57	0.0013
EPITHELIAL MESENCHYMAL TRANSITION	1.34	0.0407					1.75	0.0013
G2M CHECKPOINT	2.44	0.0008	2.01	0.0017	2.27	0.0008	2.19	0.0013
MITOTIC SPINDLE	2.00	0.0008	1.53	0.0078	1.93	0.0008	1.41	0.0404
MYC TARGETS V1	2.33	0.0008	2.60	0.0017	2.28	0.0008	2.13	0.0013
MYC TARGETS V2					1.58	0.0202		
PROTEIN SECRETION	2.11	0.0008	1.83	0.0040	1.98	0.0013	1.55	0.0368
OXIDATIVE PHOSPHORYLATION	2.21	0.0008	1.91	0.0017	1.91	0.0008	2.15	0.0013
REACTIVE OXIGEN SPECIES PATHWAY	1.55	0.0407			1.67	0.0094		
P53 PATHWAY	1.84	0.0008	1.46	0.0147	1.90	0.0008		
UV RESPONSE DN	1.36	0.0464						
UV RESPONSE UP	1.60	0.0033	1.54	0.0085	1.52	0.0072		
MTORC1 SIGNALING	1.97	0.0008	1.57	0.0054	1.93	0.0008		
PI3K AKT MTOR SIGNALING	1.82	0.0016	1.58	0.0115	1.73	0.0021		
TGF BETA SIGNALING	1.82	0.0042	1.88	0.0040	1.71	0.0054		
ADIPOGENESIS	1.64	0.0016			1.53	0.0037		
ANDROGEN RESPONSE	1.42	0.0433	1.63	0.0085				
ESTROGEN RESPONSE EARLY	1.79	0.0008	1.59	0.0043	1.71	0.0008		
ESTROGEN RESPONSE LATE	2.00	0.0008	1.74	0.0017	1.88	0.0008	1.78	0.0013
FATTY ACID METABOLISM	1.92	0.0008	1.56	0.0085	1.97	0.0008	1.57	0.0137
GLYCOLYSIS	2.10	0.0008	1.50	0.0085	2.06	0.0008	1.85	0.0013
HYPOXIA	1.59	0.0020			1.65	0.0013	1.58	0.0032
XENOBIOTIC METABOLISM	1.64	0.0016	1.43	0.0183	1.62	0.0016		
DOWNREGULATED HALLMARK PATHWAYS								
ALLOGRAFT REJECTION	−1.47	0.0079	−1.44	0.0183	−1.65	0.0014		
INTERFERON ALPHA RESPONSE	−1.95	0.0008	−2.27	0.0017	−2.27	0.0008		
INTERFERON GAMMA RESPONSE	−1.70	0.0008	−1.75	0.0017	−1.96	0.0008		
BILE ACID METABOLISM	−1.61	0.0052			−1.56	0.0074	−1.73	0.0013

All pathways presented have an FDR <  0.05. Data is presented as normalized enrichments scores (NES) where values >0 represent upregulation and values <0 represent downregulation when comparing non-responders with responders

## Discussion

We explore mechanisms underpinning the response to intranasal administration of live *L. lactis W136* in patients with refractory CRS using transcriptomics. This complements our previously reported material on the microbiome changes observed with BID administration of 1.2 billion CFU of *Lactococcus Lactis W136* directly to the nasal and sinus cavities (9). A prior study by Martensson et al. (8) reported intranasal administration of a mixture of lactobacilli derived from honeybees, no change was observed in sampled mediators nor the sinonasal microbiome. More recently, Lactobacillus administered intranasally was well tolerated by CRS subjects, there was no clinical or mechanistic assessment of results (9).

The present work uses transcriptomic methods to understand the mechanistic underpinnings of the effects of topical probiotics on the nasal and sinus surface. We describe that *L. lactis* administration is associated with differential gene expression involving biological pathways not only implicated in the regulation of immunity, but also epithelial regulation, as suggested by the identification of pathways that contribute to cell proliferation, survival signalling, and DNA damage repair. Transcriptomic changes associated with *L. lactis W136* probiotic therapy thus suggest a beneficial effect on cell cycle progression and restoration of epithelial function.

Cellular and structural integrity is essential for the appropriate function of the epithelial barrier. We have previously documented that epithelial response to wounding is impaired in CRS (9). Improvements seen in this clinical trial suggesting enhanced epithelial regeneration and repair may reflect an improvement of these processes.

We report that immune function was consistently downregulated following treatment, with decreases observed in interferon response, T cell infiltration and activation, and allograft reaction. Immune modulation is a feature of probiotics (13), thus it is not surprising to see it demonstrated here. A signal for modulation of interferon alpha and gamma signalling may reflect a reduction in the non-Type 2 component of the inflammatory CRS response (14), where persistently high levels of interferon exert detrimental effects by impairing strength and coordination of subsequent immune responses and hampering cellular function (15). These changes can contribute to the development of epithelial to mesenchymal transition characteristic of CRS (16). Moreover, high levels of Type 1 inflammatory mediators is associated with a functional defect in epithelial repair in respiratory epithelial cells (17).

This is consistent with our results. *L. lactis* administration was also associated with differential gene expression involving biological pathways implicated in epithelial regulation, as suggested by the identification of pathways that contribute to cell proliferation, survival signalling, and DNA damage repair. Transcriptomic changes associated with *L. lactis W136* probiotic therapy thus suggest a beneficial effect on cell cycle progression and restoration of epithelial function. Cellular and structural integrity is essential for the appropriate function of the epithelial barrier (18).

We have previously documented that epithelial response to wounding is impaired in CRS (19). Improvements seen in this clinical trial suggest that enhanced epithelial regeneration and repair may thus reflect an improvement of these processes.

The depth and breadth of the effects of *L. lactis W136* probiotic supplementation on cellular and epithelial function were not expected, however, these are consistent with previous reports showing improved re-epithelialisation of keratinocytes following wounding with *Lactobacillus rhamnosus* GG lysate in a scratch assay model (20) and our evolving understanding of CRS disease. The epithelial barrier plays an important role in the disease process. In addition to its role as a physical barrier to pathogens and irritants, epithelial cells are known to be responsible for initiating and coordinating defensive responses (6). As epithelial regeneration and repair are altered in patients with CRS, the increase in gene expression in multiple aspects of the cellular cycle suggests a beneficial effect of intranasal administration of *L. lactis* supplementation on the restoration of epithelial cell function and cell cycle progression. These changes were observed after only 14 days of treatment compared to the treatment length used in other studies, which is four months. Thus, the beneficial effect is expected to be greater with sustained *L. lactis* supplementation.

Taken together, these findings demonstrate that the administration of *L. lactis W136* influences multiple processes extending beyond immune regulation, suggesting effects on the epithelial barrier which can potentially modulate composition of the microbiome.

### Study limitations

This study is not a parallel group-controlled trial, and there is no direct comparison possible with saline-only irrigations. Nevertheless, in the clinical trial, saline irrigation-only use during the run-in period while was associated with aggravation of symptoms of CRS and deterioration of mucosal aspect following the withdrawal of medications. Also, the sample size is relatively small, particularly in the “CRSsNP” and “Responders” group which are smaller than the “overall population”. The pattern of response is nevertheless similar across groups and results thus should not be discounted.

In addition, there is no record of whether saline alone influences gene expression. While saline irrigations have been shown not to influence microbiome composition in CRS patients (5) this has not been assessed in a comparator trial of gene expression. However, patients had already received two weeks of saline rinse-only during the 14-day run-in period without any clinical benefit, thus it is unlikely that changes are secondary to saline alone. Lastly, changes were assessed after only 14 days of treatment, which is a short treatment period, compared to other studies in refractory CRS (21). Observed effects might thus be greater with more prolonged administration.

## Conclusion

The transcriptomic assessment suggests improvements following the application of live bacteria to the diseased sinus epithelium in refractory CRS appear to involve multiple components of the inflammation-microbiome-epithelial barrier axis implicated in CRS. *L. lactis W136* probiotic therapy influences both epithelial restoration and modulation of innate and adaptive immunity, supporting the potential interest of targeting the sinus epithelium and the microbiome as potential CRS therapies.

## Data Availability

The original contributions presented in the study are included in the article/**Supplementary Materials**, further inquiries can be directed to the corresponding author.

## Take Home Message

Transcriptomic profiling suggests effects of live probiotic Lactococcus lactis W136 bacterium to the diseased sinus epithelium in refractory CRS involve not only modulation of Type 1 inflammation and microbiome but also implicate restoration of the integrity of the epithelial barrier axis.

## Supplementary material

***Supplementary figure S1. Transcriptomic assessment of response to intranasal Lactococcus lactis W136 in all CRS patients.*** Heatmap of top 50 differentially expressed genes with an FDR < 0.05.

***Supplementary figure S2. Transcriptomic assessment of response to intranasal Lactococcus lactis W136 in the chronic sinusitis with nasal polyps (CRSwNP) subgroup.*** Heatmap of the top 50 differentially expressed genes with an FDR < 0.05.

***Supplementary figure S3. Transcriptomic assessment of response to intranasal Lactococcus lactis W136 i**n **the chronic sinusitis without nasal polyps (CRSsNP) subgroup.*** Heatmap of the top 50 differentially expressed genes with an FDR < 0.05.

***Supplementary figure S4. Transcriptomic assessment of response to intranasal Lactococcus lactis W136 in the responders subgroup.*** Heatmap of the top 50 differentially expressed genes with an FDR < 0.05.

***Supplementary figure S5. Transcriptional response to 14 days of Lactococcus lactis W136.*** Gene Set Expression Analysis (GSEA) of response to treatment. (**a**) All CRS patients, (**b**) chronic sinusitis with nasal polyps (CRSwNP), (**c**) chronic sinusitis without nasal polyps (CRSsNP), (**d**) Responders. Differential expression in selected significant pathways demonstrate a consistent pattern of enhanced epithelial renewal (E2F TARGETS, EPITHELIAL MESENCHYMAL TRANSITION, G2M CHECKPOINT, MYC TARGETS V1, MYC TARGETS V2, PROTEIN SECRETION, MITOTIC SPINDLE), Increased DNA repair (P53 PATHWAY, UV RESPONSE DN, UV RESPONSE UP), reactive oxygen species (ROS) regulation (HYPOXIA, REACTIVE OXIGEN SPECIES PATHWAY, OXIDATIVE PHOSPHORYLATION), activation of signalling pathways (PI3K AKT MTOR SIGNALING, MTORC1 SIGNALING, TGF BETA SIGNALING) and downregulation of adaptive and innate immune signalling (ALLOGRAFT REJECTION, INTERFERON ALPHA RESPONSE, INTERFERON GAMMA RESPONSE). All pathways presented have an FDR < 0.05. Data is presented as normalized enrichments scores (NES) where values > 0 represent upregulation and values < 0 represent downregulation when comparing non-responders with responders.
