# Supplementary figures and images for: Response to intranasal Lactococcus lactis W136 probiotic supplementation in refractory CRS is associated with modulation of non-type 2 inflammation and epithelial regeneration
Source: Front Allergy. 2023 Mar 15;4:1046684. doi: 10.3389/falgy.2023.1046684 (PMC10050565; doi:10.3389/falgy.2023.1046684)

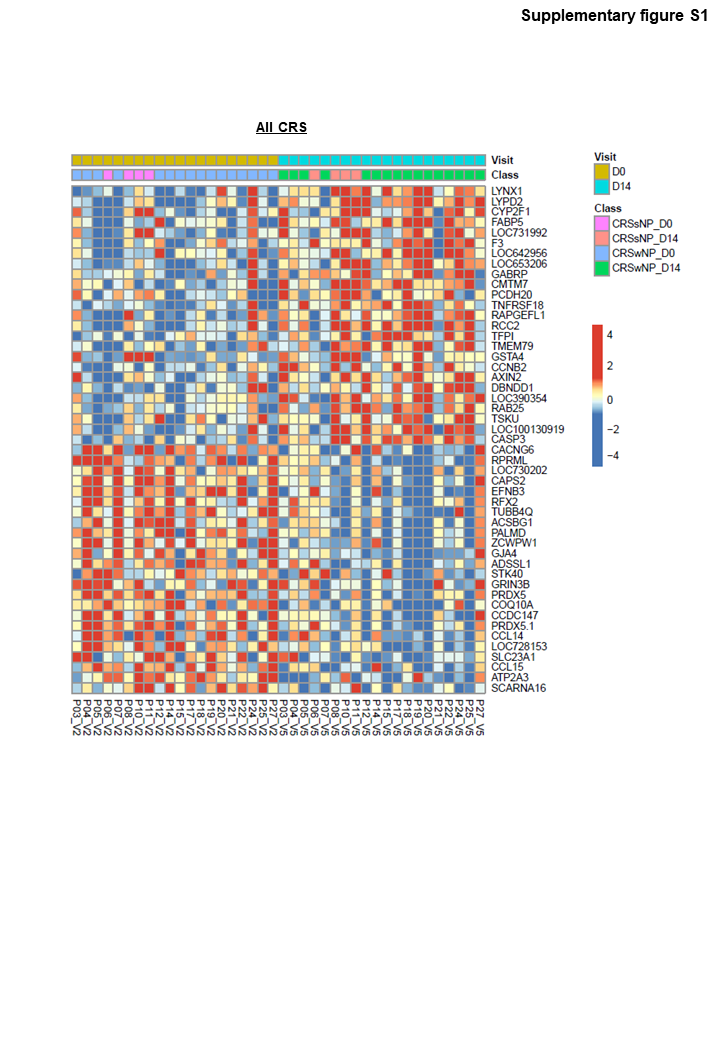

Supplement: Supplementary file 1 [file Image1.tif]

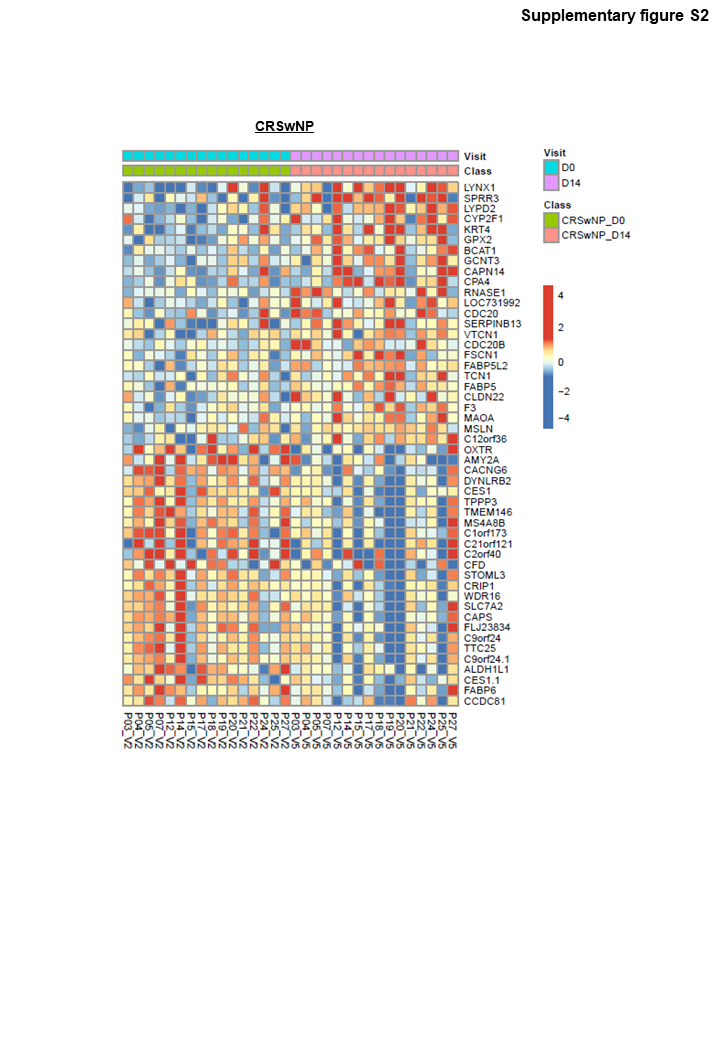

Supplement: Supplementary file 2 [file Image2.tif]

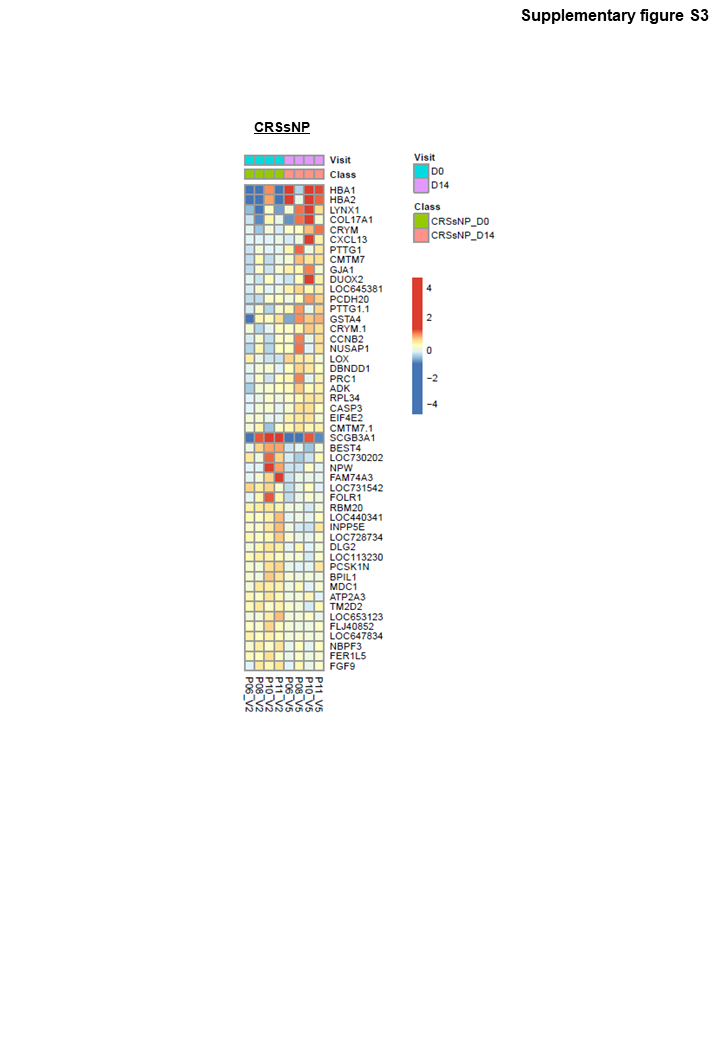

Supplement: Supplementary file 3 [file Image3.tif]

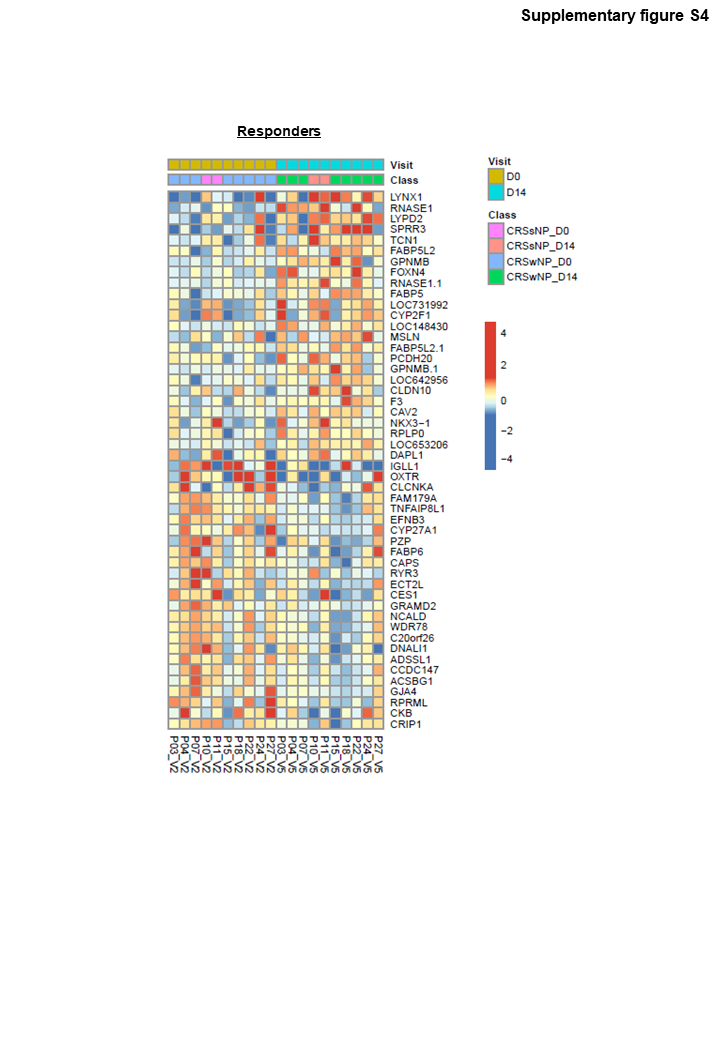

Supplement: Supplementary file 4 [file Image4.tif]

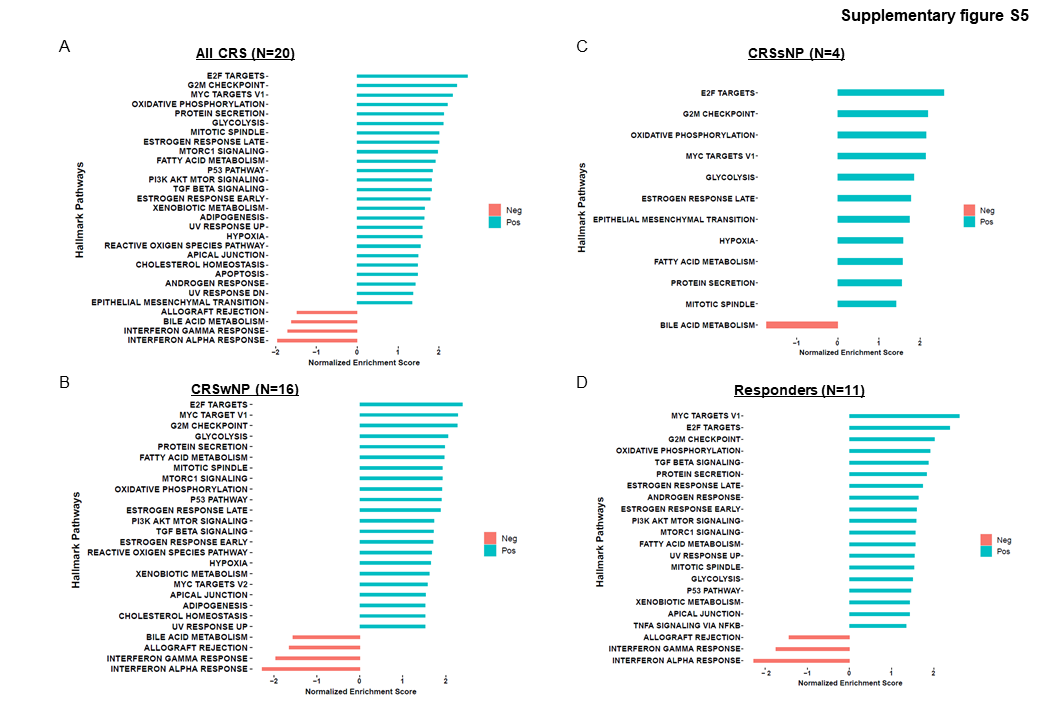

Supplement: Supplementary file 5 [file Image5.tif]
